# In vivo examination of pathogenicity and virulence in environmentally isolated *Vibrio vulnificus*

**DOI:** 10.1002/mbo3.1427

**Published:** 2024-07-23

**Authors:** Shannon E. Pipes, Charles R. Lovell, Katie L. Kathrein

**Affiliations:** ^1^ Department of Biological Sciences University of South Carolina Columbia South Carolina USA

**Keywords:** emerging infectious disease, environmental pathogens, genomes, pathogenesis, *Vibrio vulnificus*, Zebrafish

## Abstract

Human exposure to *Vibrio vulnificus*, a gram‐negative, halophilic environmental pathogen, is increasing. Despite this, the mechanisms of its pathogenicity and virulence remain largely unknown. Each year, hundreds of infections related to *V. vulnificus* occur, leading to hospitalization in 92% of cases and a mortality rate of 35%. The infection is severe, typically contracted through the consumption of contaminated food or exposure of an open wound to contaminated water. This can result in necrotizing fasciitis and the need for amputation of the infected tissue. Although several genes (*rtxA1, vvpE*, and *vvhA*) have been implicated in the pathogenicity of this organism, a defined mechanism has not been discovered. In this study, we examine environmentally isolated *V. vulnificus* strains using a zebrafish model (*Danio rerio*) to investigate their virulence capabilities. We found significant variation in virulence between individual strains. The commonly used marker gene of disease‐causing strains, *vcgC*, did not accurately predict the more virulent strains. Notably, the least virulent strain in the study, *V. vulnificus* Sept WR1‐BW6, which tested positive for *vcgC, vvhA*, and *rtxA1*, did not cause severe disease in the fish and was the only strain that did not result in any mortality. Our study demonstrates that virulence varies greatly among different environmental strains and cannot be accurately predicted based solely on genotype.

## Graphical abstract

*Vibrio vulnificus* poses an emerging threat as a human pathogen. While the incidence of human disease from *V. vulnificus* has historically been low, rising water temperatures are leading to an increase in human exposure across various latitudes. The severity of infections caused by *V. vulnificus* is particularly alarming, with most cases requiring hospitalization and a mortality rate of 35%. In this study, we investigate several strains of *V. vulnificus* isolated from the environment and find that their levels of pathogenicity vary. Interestingly, the genotypes of these strains do not align completely with previously identified markers of virulence.

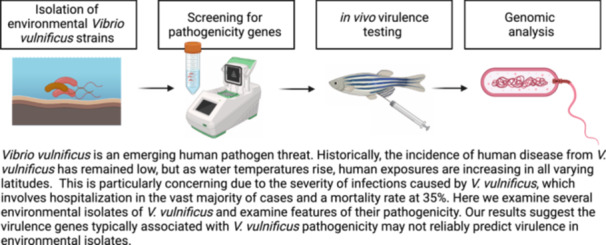

## 1 INTRODUCTION

*Vibrio vulnificus* is a gram‐negative, halophilic bacterial species that is found in estuarine and coastal waters. This naturally occurring environmental human pathogen is a great concern for public health due to its routine isolation from the water column, sediment, and shellfish. There are roughly 80,000 cases of *Vulnificus* sp*.* infections, or vibriosis, each year in the United States. Although the number of infections caused by *V. vulnificus* is a relatively low percentage of total vibriosis cases, the severity of injection makes *V. vulnificus* a formidable threat (Scallan et al., 2011). Most *V. vulnificus* cases originate from wound infections and septicemia, with only 5% involving gastroenteritis (Scallan et al., 2011). Hospitalization rates for *V. vulnificus* infection are high, in 92% of cases, and the mortality rate is 35% (Scallan et al., 2011). If infection occurs through an open wound, *V. vulnificus* infection can lead to sepsis and necrotizing fasciitis. The mortality rate of *V. vulnificus* when it invades the bloodstream increases to 60%. In seafood‐related deaths in the United States, *V. vulnificus* is the major cause, responsible for 95% of these infections. Despite the severity and concern with *V. vulnificus* infections, it is still poorly understood which genes are directly involved in pathogenicity (Al‐Assafi et al., 2014; Linkous & Oliver, 1999).

Understanding the virulence of *V. vulnificus* has been underway for several years, yet little is known about the pathogenicity mechanism or which specific genes are involved in virulence for this species. Several strains can produce cytotoxic effects in human epithelial cell lines, but research has heavily focused on the strains from clinically isolated sources (Hiyoshi et al., 2010; Raimondi et al., 2000). Little work has been done to test the virulence capabilities of environmentally derived strains, based on the premise that few strains that naturally persist in the environment cause serious disease, or occur at low, infrequent incidences (Baker‐Austin et al., 2008; DePaola & Kaysner, 2004). However, when environmentally derived strains of *V. vulnificus* and *Vibrio parahaemolyticus* were compared to clinical strains using a human gastrointestinal epithelial cell line, both clinically and environmentally isolated strains caused similar degrees of damage to human cells in vitro (Klein, 2018).

There have been implicated virulence genes proposed to contribute to the pathogenicity process of the organism, but unlike its relative *Vibrio cholerae*, there is still no defined pathogenicity mechanism*. V. vulnificus* is commonly broken down into groups based on biotypes based on their biological and biochemical properties: biogroup 1, biogroup 2, and biogroup 3 (Linkous & Oliver, 1999; Oliver, 1989). Biogroup 1 strains are derived most frequently in the clinic, from patients who are actively suffering from a *V. vulnificus* infection. Biotype 2 is an extremely rare human pathogen isolated from diseased eels (Amaro & Biosca, 1996). A more recent addition to the clade of *V. vulnificus* is Biotype 3, which has thus far only been isolated in Israel (Efimov et al., 2013). The genome of Biotype 3 is more closely related to the Biotype 1 genomes, with 90% similarity, whereas the Biotype 2 genome has 87% similarity. Previous studies have suggested that pathogenesis occurs through the penetration of cells by extracellular proteins released by the invading bacteria, which causes tissue damage, especially to tissue of vascular nature (Al‐Assafi et al., 2014; Jeong & Satchell, 2012).

*V. vulnificus* is also commonly broken down into two genotypes. These two groups are established by the presence of a specific *virulence‐correlated gene* (*vcg*), either type E or type C. Biotype 1 strain contains the *vcgC* gene (Rosche et al., 2005). The vast majority (90%) of clinically isolated strains of *V. vulnificus* contain the *vcgC* variant, while the vast majority of environmentally isolated strains contain *vcgE* (87%) (Rosche et al., 2005). Despite this, the *vcg* gene likely does not contribute to virulence as it does not code for a protein. Furthermore, many environmental isolates of *V. vulnificus* also contain the *vcgC* variant, suggesting that the presence of *vcg* may not be an accurate way of predicting virulence in the environmental setting (Klein, 2018).

Although no mechanism has been defined, several genes have been identified as possible virulence factors in *V. vulnificus*. The hemolysins (*vvhA* and *vvhB*), toxins (*rtxA1*), siderophores, outer membrane proteins and lipopolysaccharides (LPS), and flagella components have also been implicated in *V. vulnificus* virulence (Goo et al., 2006; Jeong & Satchell, 2012; Jones & Oliver, 2009; Kim et al., 2010, 2012; Yokochi et al., 2013). The gene *vvhA* encodes a hemolysin that induces cytolysis and death by making pores in erythrocyte cell membranes (Kim et al., 2010). Hemorrhagic damage and skin necrosis are triggered by the protease *vvpE*. This results in vascular permeability, causes edema, and is ultimately lethal in mouse models of infection (Kothary & Kreger, 1987). The *VvpE* gene has also been implicated in the invasiveness of *V. vulnificus.* The protein encoded by *VvpE* may induce proteolytic cleavage of lactoferrin and IgA antibodies (Kim et al., 2007). The *rtxA1* gene is one of the most characterized virulence factors of *V. vulnificus* (Kim et al., 2016). This gene is a cytotoxin with multiple functions, triggering rearrangement of the cytoskeleton, hemolysis, cytotoxicity, and facilitating tissue invasion, which can ultimately lead to lethality in infected mice. Despite this work, the exact mechanism of *V. vulnificus* virulence remains unresolved.

To address variation in the virulence of environmental isolates of *V. vulnificus*, we utilized a Zebrafish model. Zebrafish (*Danio rerio*) have successfully been used to study the virulence and pathogenic capabilities of many different types of bacteria, including several *Vibrio* species (Bergeron et al., 2017; Mitchell & Withey, 2018; Nag et al., 2020; Neely et al., 2002; Paranjpye et al., 2013). Although Zebrafish have a lower internal body temperature than mammalian model systems, which may result in slower bacterial growth, the Zebrafish model has been shown to fully recapitulate the full life cycle of bacterial infection (Mitchell & Withey, 2018). Zebrafish have innate and adaptive immune systems, so the symptomatic effects of injection are nearly identical to humans (Da'as et al., 2011; Stemple & Driever, 1996; Sullivan & Kim, 2008). Here, we utilized the Zebrafish model to understand how the virulence of environmentally isolated *V. vulnificus* strains compares to clinically isolated strains. We discovered a range in virulence, which differs greatly between strains, and our data suggest that the use of *vcgC* and *vcgE* as potential predictors of genotype and virulence may not be a reliable tool.

## 2 MATERIALS AND METHODS

### 2.1 Strain isolation

Environmental *V. vulnificus* strains were isolated from lower salinity waters in Winyah Bay and the Waccamaw River near Georgetown, SC, USA (33°20′ N, 79°12′ W) by Daniel Tufford. CHROMagar *Vibrio* (DRG International) was used for the initial plating of the water samples to isolate *V. vulnificus* strains (following the US Food and Drug Administration protocol) (DePaola & Kaysner, 2004). After this initial isolation, *V. vulnificus* strains were routinely cultivated on saline lysogeny agar (SLA; per L; 10 g tryptone, 5 g yeast extract, 15 g NaCl, 15 g Bacto Agar) and TCBS agar (Thiosulfate Citrate Bile Salts Sucrose Agar), a selective and differential growth medium specific to *Vibrio* spp., which is recommended to use for the selective isolation and cultivation of *Vibrio* spp. from clinical specimens (Hardy Diagnostics). *V. vulnificus* strain JY1701 was isolated environmentally via methods described by Rosche et al. (2005).

### 2.2 Whole genome sequencing

Genomic DNA was isolated using a DNA purification kit following the manufacturer's protocol for Gram‐negative organisms (Wizard Genomic DNA Purification kit, Promega). After extraction, the DNA was quantified via Quibit fluorimetry and libraries were prepared. *V. vulnificus* Aug‐WR2‐BW was sequenced using an Illumina MiSeq (V3 26300 base) at the Indiana University Center for Genomic Studies. The work was conducted in conjunction with the Genome Consortium for Active Teaching NextGenSequencing Group (GCAT‐SEEK) shared run (Buonaccorsi et al., 2011, 2014). After sequencing, reads were: filtered (median phred score 0.20), trimmed (phred score 0.16), and assembled using the paired‐end de novo assembly option in NextGENe V2.3.4.2 (SoftGenetics). The Rapid Annotation with Subsystem Technology (RAST) web service (Aziz et al., 2008; Overbeek et al., 2014) was used for assembly improvement, analysis, and guided contig reordering. Dotplot comparisons were used for genome alignment. Whole genome sequence data generated through this study was submitted to the NCBI GenBank (accession number: GCA_003798485.1).

### 2.3 Zebrafish husbandry and care

Zebrafish (*D. rerio*) used in this study were from the Tübingen strain. Maintenance and breeding were conducted in accordance with the Association for Assessment and Accreditation of Laboratory Animal Care (AAALAC) guidelines, which included housing zebrafish in reverse osmosis‐filtered water‐flow tanks maintained at 28.5°C. Zebrafish were fed a commercial diet (Skretting) once per day. The salinity of zebrafish water was maintained at 0.5 g/L. Conditions used for virulence studies are described below.

### 2.4 Bacterial growth conditions

Table 1 contains the strains of bacteria used in this study. Overnight cultures of strains were grown in a medium containing 15 ppt NaCL SLA broth using a shaking incubator at 37°C. Two PBS washes were conducted on cultures before inoculation. We performed serial 10‐fold dilutions of the cultures before plating on 15 ppt NaCl SLA to confirm the concentration of the inoculum. Controls for this experiment included PBS buffer as well as a non‐virulent strain of *Vibrio*, *Vibrio pacinii* DSM 19139^T^. *V. pacinii* was grown overnight in 15 ppt NaCl SLA broth at 23°C.

**Table 1:** Gene distribution of *Vibrio vulnificus* strains used in zebrafish inoculations.

	Genes screened via PCR				
	*vvhA*	*vcgE*	*vcgC*	*rtxA1*	*vvpE*
*V. vulnificus* strains					
**ATCC 27562** ^**T**^	+	−	+	+	+
**ATCC BAA‐86**	+	−	+	+	+
**ATCC 33817**	+	−	+	+	+
JY1701	+	+	−	+	−
June BR1‐BW3	+	+	−	+	+
Aug WR2‐BW	+	+	−	+	+
Aug 05‐21 BW1	+	+	−	+	+
Sept WR4‐BW1	+	+	−	−	−
Oct WR3‐SW	+	+	−	+	+
Oct WR1‐SW	+	+	−	+	+
Oct 05‐25‐BW	+	+	−	+	−
Aug WR1‐BW6	+	−	+	+	−
Oct 05‐20‐BW	+	−	+	+	+
June WR3‐SW	+	+	+	+	+
June 05‐25‐SW1	+	+	+	+	+
Aug 05‐25‐BW3	+	+	+	+	+
Sept 05‐20‐BW4	+	+	+	+	+

*Note*: Bold indicates strains from a clinical source. Superscript T indicates type strain.

### 2.5 PCR virulence gene screening

After overnight incubation at 37°C in SLB, isolates were centrifuged and transferred to sterilized distilled water, where DNA was extracted through boiling at 95–100°C for 20 min. For all PCR reactions, 1 μL of the sample was used in each reaction. The species of the bacteria was confirmed by amplification of the *recombinase A* (*recA*) gene (Thompson et al., 2005). A common housekeeping gene that is typically used for species identification is the 16s rRNA gene; however, in vibrios, this gene is too highly conserved and does not allow for species resolution (Thompson et al., 2004). Amplicons were sent off for sequencing (Eurofins) and a phylogenetic tree using comparison sequences from NCBI was created. The protocol and primers that were used followed the protocols outlined by Thompson (2005). PCR products for recA (790 bp) were separated on a 1.5% agarose gel. The ABI Prism 3730 DNA analyzer was used for sequencing. Once sequences were received, they were edited, and the Kimura 2 parameter model with Mega 7 was used to make the maximum‐likelihood phylogenetic trees (Kumar et al., 2016; Tamura et al., 2016).

*V. vulnificus* strains were screened for the following virulence factors: *vvhA*, *vcgC*, *vcgE*, *vvpE*, and *rtxA1*. The virulence correlated genes (*vcgC* and *vcgE*) variants were used as indicator genes to differentiate between strains thought as avirulent (*vcgE* positive) and pathogenic (*vcgC* positive). PCR primers of Warner and Oliver (2008b) were used to amplify *vvhA* (410 bp), *vcgE* (199 bp), and *vcgC* (97 bp). The primers of Liu et al. (2007) were used to amplify the *rtxA1* gene and the primers of Jeong et al. (2001) were for the *vvpE* (697 bp) gene. PCR products for all virulence genes were separated on a 1.5% agarose gel.

### 2.6 Intraperitoneal challenge

For challenge experiments, zebrafish were anaesthetized in 25 mg/L tricaine. Once fully anaesthetized, zebrafish were removed from the anesthesia tank and placed on a 25 mg/L tricaine‐soaked sponge for the duration of the injection procedure. Fish were challenged using an intraperitoneal (IP) injection of 10 μl of the inoculum using a 33‐gauge needle on a Hamilton syringe (Hamilton). The needle was placed at the midway point located between the pectoral fin and the anus in accordance with previously published protocols (Lefebvre et al., 2009; Paranjpye et al., 2013). After injection, Zebrafish were housed in off‐system individual glass aquariums, with water supplied from the system water of the zebrafish facility. Water temperature was maintained at room temperature or slightly above (26–28°C). A lethal dose of tricaine was used to sacrifice any surviving fish at the experimental endpoint of 3 days. All aquariums and water were disinfected with 10% bleach solution.

### 2.7 Virulence evaluation and statistical analysis

Each strain was tested on three fish per trial, and each trial was repeated two times. Fish were monitored for 7 h post initial IP injection. Fish were housed for 3 days with monitoring, for several hours each consecutive day. Fish were viewed grossly to determine any signs of external injury, such as swelling, redness, and lesions, an increase in fecal production, changes in swimming patterns, changes in breathing patterns, and death. To determine how the bacteria infiltrate the fish body, some fish were sectioned into three regions: head, abdomen, and tail regions. These sections were weighed, homogenized, and then resuspended into 900 microliters of PBS. These were then serially diluted 10‐fold and were plated onto TCBS agar to re‐isolate the *Vibrio* bacteria to get colony counts and determine the concentration of *Vibrio* cells per gram of fish tissue per region. A one‐way analysis of variance (ANOVA) using SPSS was performed comparing the means of fish lethality for each clade of tested *Vibrio* strains: *vcgC*‐positive, *vcgE*‐positive, and both *vcgC* and *vcgE*‐positive. The significance level for the test was set at a *p*‐value of 0.05. A one‐way ANOVA was also used to compare the means of fish lethality between the clinically isolated strains and the environmentally isolated strains. A significance level of 0.05 was also used.

### 2.8 Genome gazing

*V. vulnificus* WR2‐BW, JY1701, and 27562^T^ genomes were used to genome gaze and compare the differences in gene makeup between the three strains. The RAST SEED Viewer application was used to compare the genomes of the three strains, which were compared using a sequence‐based function (Aziz et al., 2008; Overbeek et al., 2005, 2014). The comparison tables were downloaded and using the most virulent strain from the three of them as the reference organism, gene profiles of missing genes from the two less virulent strains were categorized.

## 3 RESULTS

**Figure 1:**
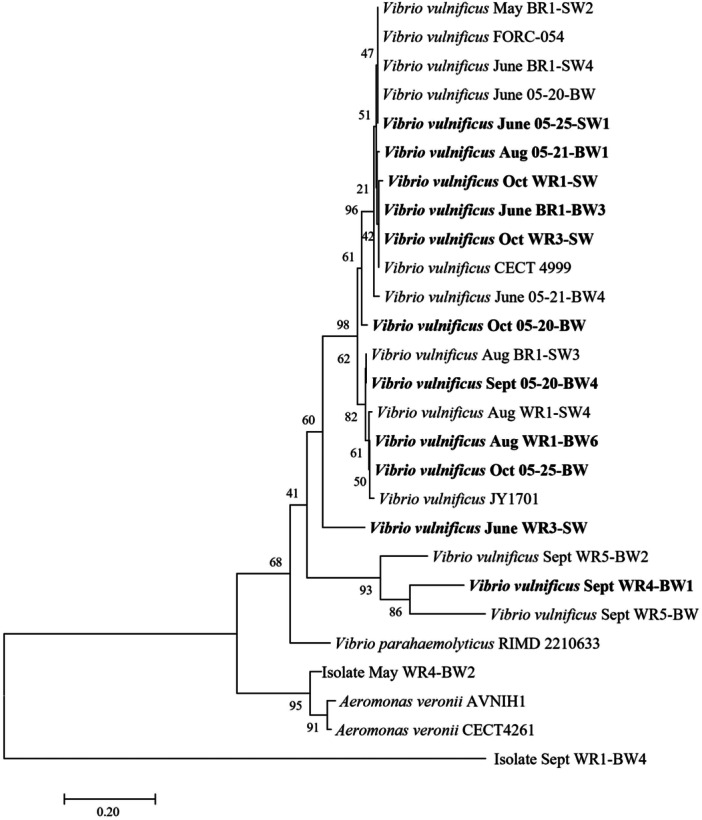
Maximum‐likelihood phylogeny of *recA* gene sequences (Kimura 2‐parameter model). The bootstrap values represent 1000 replications. NCBI GenBank was the source for the acquisition of the reference sequences. Bolded sequences are the environmental strains used in this study.

Several environmental *V. vulnificus* strains were isolated from the lower salinity waters of the Waccamaw River and the Winyah Bay areas near Georgetown, SC, USA. Gene sequences of the *recA* gene were used to confirm that the environmentally isolated bacterial species were *V. vulnificus*, based on the phylogeny and percent similarity identity scores compared to reference genes of the *recA* gene in confirmed and sequenced strains of *V. vulnificus* from NCBI GenBank (Figure 1). For all species confirmed to be *V. vulnificus* used in the study, 100% tested positive for containing the *vvhA* gene. Of the 13 environmentally isolated strains used, seven strains (54%) were *vcgE* variant positive, two strains (15%) were *vcgC* variant positive, and four strains (30%) contained both variants of the *vcg* gene. For the *vgcE*‐positive strains, all but one contained the *rtxA1* gene, and five of the seven were positive for *vvpE*. Strain Sept WR1‐BW4 was the only strain of the *vgcE* clade that was negative for *rtxA1* and it was also negative for *vvpE*. The other strain negative for *vvpE* was Oct 05‐25‐BW. Looking at the *vcgC* clade, strain Oct SF 05‐20‐BW tested positive for both *rtxA1* and *vvpE*, whereas strain Aug WR1‐BW6 tested positive for only *rtxA1*. All strains that contained both *vcg* variants also contained *rtxA1* and *vvpE* (Table 1).

**Figure 2:**
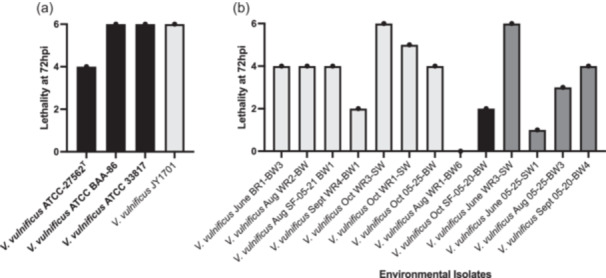
Lethality rate of fish totaled from two independent trials of *Vibrio vulnificus* strains. Clinically or independently isolated strains (a) include ATCC‐27562^T^, ATCC‐BAA‐86, ATCC 33817, and JY1701. All other strains are environmentally isolated for this study (b). Controls for the experiment included fish injected with PBS buffer and an avirulent *Vibrio* species, *Vibrio pacinii*. Black bars indicate *vcgC*‐positive strains. Light gray bars indicate *vgcE*‐positive strains. Dark gray bars indicate strains positive for both *vcgC* and *vcgE*.

To examine virulence, three fish per strain were injected into the abdomen with 10 µL of bacterial culture with a cell concentration of 10^3^ CFUs/mL. Virulence varied greatly between individual strains (Figure 2). The most common early symptoms in the fish included diarrhea and site injection redness and irritation. As the infection progressed, many fish displayed difficulty swimming and maintaining positive buoyancy, signs of labored breathing, and many became lethargic, lying prone on the bottom of the aquarium. In the more virulent strains, there was enterohemorrhagic activity, and upon dissection, many fish that succumbed early in the infection had a lower blood volume and visible tissue damage when compared to fish that survived the entire trial. A total of 102 fish were used in this study (17 strains, two trials for each strain, each trial involving three inoculated fish). Out of the total study, 67 (65%) fish succumbed as a direct result of the *V. vulnificus* infection (Table 2). For the environmentally isolated strains, 58% of the fish directly succumbed to the *V. vulnificus* infection (Table 2)*.* For the clinically isolated strains, 91% of the inoculated fish succumbed to the bacterial infection. The clinically isolated strain ATCC BAA‐86 was the most virulent of all tested strains, where both trials with this organism resulted in 100% mortality within 24 h of exposure. Comparatively, the least virulent strain was *V. vulnificus* strain Aug WR1‐BW6, which was not lethal to any fish tested in the two trials, and the fish exhibited very little symptomatic response to the bacterial injection. Inoculation with *Vibrio pacinii* DSM 19139^T^, an avirulent bacterial species, and PBS alone were used as controls for injections. No lethality, illness, or distress was observed in these treatment groups.

**Table 2:** Fish lethality rate of *Vibrio vulnificus* strains used in zebrafish inoculations.

Fish lethality rate					
*V. vulnificus* strains tested	12 h (dead/total tested)	24 h.	48 h.	72 h.	Total fish that died during the trial
**ATCC‐27562** ^**T**^	0/6	1/6	2/6	4/6	4
**ATCC BAA‐86**	1/6	6/6	—	—	6
**ATCC 33817**	0/6	5/6	6/6	—	6
JY1701	1/6	5/6	6/6	—	6
June BR1‐BW3	0/6	0/6	3/6	4/6	4
Aug WR2‐BW	0/6	2/6	4/6	4/6	4
Aug SF‐05‐21 BW1	0/6	4/6	4/6	4/6	4
Sept WR4‐BW1	0/6	0/6	1/6	2/6	2
Oct WR3‐SW	0/6	0/6	6/6	—	6
Oct WR1‐SW	0/6	0/6	2/6	5/6	5
Oct 05‐25‐BW	0/6	0/6	3/6	4/6	4
Aug WR1‐BW6	0/6	0/6	0/6	0/6	0
Oct SF‐05‐20‐BW	0/6	0/6	0/6	2/6	2
June WR3‐SW	0/6	4/6	5/6	6/6	6
June 05‐25‐SW1	0/6	1/6	1/6	1/6	1
Aug 05‐25‐BW3	0/6	0/6	0/6	3/6	3
Sept 05‐20‐BW4	0/6	0/6	2/6	4/6	4

*Note*: Totals come from two independent trials of each strain. Each trial was repeated twice. Bold indicates strains from a clinical source. Superscript T indicates type strain.

A one‐way ANOVA statistical test was completed to compare the means of lethality between the three clades of tested strains: *vcgE*‐positive, *vcgC*‐positive, and both variant positive, based on the hypothesis that the means between the different gene clades would produce a difference in mean lethality during the trials. This analysis showed no statistically significant difference in the means between the three groups, with a *p*‐value of 0.687. The one‐way ANOVA looking at the means of lethality between the clinically isolated strains and the environmentally isolated strains also showed no significant difference between the two, with a *p*‐value of 0.051.

Fish that were injected with strains BAA‐86 and 05‐21‐BW1 were used to determine cell recovery per gram of tissue in three sections of the fish: head, abdomen, and tail. These fish regions were homogenized and serially diluted onto TCBS agar. For BAA‐86, the results showed that the highest recovery of bacteria came from the abdomen region, with the average of six fish being 1.58 × 10^9^ CFUs g^−1^, the tail region with the next highest concentration at 1.07 × 10^8^ CFUs g^−1^, and the head region with the lowest at 2.35 × 10^7^ CFUs g^−1^. The results for environmental strain 05‐21‐BW1 held consistent when compared to BAA‐86 with the abdomen region with the highest cells recovered per gram of tissue at 1.55 × 10^9^ CFUs g^−1^, but for the environmental strain, the head had the next highest cells recovered at 5.65 × 10^7^ CFUs g^−1^, and the tail region with the lowest at 8.18 × 10^6^ CFUs g^−1^.

Of the three strains with full genome sequences (Accession numbers for *V. vulnificus* JY1701, *V. vulnificus* ATCC 27562^T^, and *V. vulnificus* WR2‐BW, respectively: AFSY00000000, NZ_AMQV00000000, GCA_003798485.1), *V. vulnificus* JY1701 had the highest level of virulence, with 100% lethality. Both strains ATCC 27562^T^ and WR2‐BW resulted in 67% lethality. JY1701 and WR2‐BW are *vcgE*‐positive strains, whereas ATCC 27562^T^ is a *vcgC*‐positive strain (Table 1). xJY1701 was set as the reference genome for comparative genome analysis across the strain due to the high virulence of this strain. There were a total of 305 unique genes present in JY1701 that were not present in either WR2‐BW or ATCC 27562^T^. Only 47 of those 305 genes have a known function (Table 3). The rest of the unique genes (258 genes, or 84.5% of the total unique genes) were hypothetical proteins, genes of unknown function, or phage‐related genes. Four candidate virulence‐factor‐associated genes were identified in *V. vulnificus* JY1701 that were not present in WR2‐BW and ATCC 27562^T^. They are T1SS‐secreted agglutinin RTX, a virulence‐associated E gene, a putative integrase, and a WzxE protein. These genes may represent previously unknown virulence factors in *V. vulnificus.*

**Table 3:** Results from genome gazing at the genes present and unique to strain *Vibrio vulnificus* JY1701 and not present in strains *V. vulnificus* 27562^T^ or WR2‐BW.

Gene number in *V. vulnificus* JY1701	Length of gene (No. amino acids)	Function of gene
424	53	Type cbb3 cytochrome oxidase biogenesis protein CcoI; Copper‐translocating P‐type ATPase (EC 3.6.3.4)
479	210	TonB‐dependent receptor
480	483	TonB‐dependent receptor
483	147	Putative membrane protein
520	46	Maltoporin (maltose/maltodextrin high‐affinity receptor, phage lambda receptor protein)
615	41	2‐oxoglutarate dehydrogenase E1 component (EC 1.2.4.2)
689	152	Rhs family protein
1138	239	Beta‐1,4‐galactosyltransferase
1141	393	UDP‐Bac2Ac4Ac hydrolyzing 2‐epimerase NeuC homolog
1143	213	4‐amino‐6‐deoxy‐N‐Acetyl‐d‐hexosaminyl‐(Lipid carrier) acetyltransferase
1149	77	Acyl carrier protein, putative
1152	672	Acyl protein synthase/acyl‐CoA reductase RfbN
1153	131	Acyl protein synthase/acyl‐CoA reductase RfbN
1155	325	Polysaccharide deacetylase
1171	141	S‐adenosylhomocysteine hydrolase
1175	135	Structural protein P5
1178	157	Methyl‐accepting chemotaxis protein
1179	124	Mg‐dependent DNase
1183	278	Outer membrane receptor protein
1446	51	Neopullulanase (EC 3.2.1.135)
1791	266	Arginine/ornithine antiporter ArcD
2057	65	Lipid carrier: UDP‐N‐acetylgalactosaminyltransferase (EC 2.4.1.‐)/Alpha‐1,3‐N‐acetylgalactosamine transferase PglA (EC 2.4.1.‐); Putative glycosyltransferase
2061	388	Glycosyl transferase, group 1
**2063**	**417**	**WzxE protein**
2215	931	Chromosome segregation ATPases
2267	587	DNA double‐strand break repair Rad50 ATPase
2284	290	EF‐hand domain protein
2623	738	Translation‐disabling ACNase RloC
2674	118	ORF2
2991	637	DNA helicase II‐related protein
3371	288	**Putative integrase**
**3374**	**420**	**Virulence‐associated E**
3459	297	Type III restriction‐modification system methylation subunit (EC 2.1.1.72)
3460	330	Type III restriction‐modification system methylation subunit (EC 2.1.1.72)
3461	799	Type III restriction‐modification system DNA endonuclease res (EC 3.1.21.5)
3636	38	Trehalose‐6‐phosphate hydrolase (EC 3.2.1.93)
3686	264	Putative alpha‐dextrin endo‐1, 6‐alpha‐glucosidase
3890	161	GCN5‐related N‐acetyltransferase
3967	40	Alcohol dehydrogenase (EC 1.1.1.1)
4081	176	Predicted transcriptional regulator
4082	302	Predicted nucleotide‐binding protein
4259	445	Articulin, putative
4314	265	Putative type II restriction endonuclease
4334	141	Putative glyoxalase
4402	224	HAD superfamily hydrolase
4425	159	Putative acetyltransferase
**4461**	**79**	**T1SS secreted agglutinin RTX**

*Note*: The genes in this table are genes with known functions. Hypothetical proteins, genes with unknown function, or phage‐related genes are not included. Bolded genes indicate genes that may have a possible virulence‐related function.

## 4 DISCUSSION

The pathogenicity of *V. vulnificus* is complex, undefined, and varied across strains. Here, we show a complicated mechanism for *V. vulnificus* virulence, where virulence between those isolated from the environment differs greatly and does not necessarily correlate with the presence of previously implicated virulence‐related genes: *vvhA* and *vvhB*, *rtxA1*, and *vvpE.* When examined in our system, the clinically isolated *vcgC*‐positive strains were highly virulent in the zebrafish. Yet, the presence of *vcgC* in our study did not always reflect the highest virulence capabilities. The least virulent strains from the study were *V. vulnificus* Sept WR1‐BW6, June 05‐25‐SW1, and Sept 05‐20‐BW4, all of which contain a copy of the *vcgC* gene. Our study included seven strains lacking *vcgC* and positive for *vcgE* alone. Of those, six of the seven strains were as virulent as the least virulent clinically isolated strain of *V. vulnificus* (ATCC 27562^T^), where each of these strains resulted in 67% mortality.

Previous studies have shown that *V. vulnificus* isolates from oysters show an overwhelming proportion of *vcgE*‐positive strains, and this has been considered a reason why the incidence of *V. vulnificus* infections is relatively low; if *vcgC* strains are not predominant, then it is less likely for a person to consume or encounter “virulent strains” (Warner & Oliver, 2008a). There may be an important ecological reason as to why the *vcgE* genotype does predominate in oysters. However, this genotyping protocol implies that the *vcgE* strains are incapable of causing disease. Our study demonstrates that there are virulent *vcgE* strains. In addition, cytotoxicity trials in previous studies have shown that *vcgE* strains are capable of destroying epithelial cells and causing disease (Klein, 2018). The introduction of live bacteria into zebrafish is not a trigger of death as a process, as not a single fish that was inoculated with *Vibrio pacinii* DSM 19139^T^, an avirulent bacterial species, died or showed any signs of illness or distress. Relying on genotype alone (*vcgC* vs. *vcgE*) as a marker for pathogenic versus nonpathogenic status may not be reliable.

Through genome gazing of published data sets from tested strains of *V. vulnificus*, it was determined that 305 genes were unique to *V. vulnificus* JY1701 when compared to *V. vulnificus* ATCC 27562^T^ and Aug WR2‐BW. Of those 305 genes, only 47 have a known or defined function. The remaining genes are hypothetical, undefined, or phage‐related. We identified four genes that may contribute to virulence‐related function. Those genes include the T1SS‐secreted agglutinin *RTX*, *wzxE*, *virulence‐associated E*, and a putative integrase.

The Type 1 secretion systems (T1SS) contain genes present in many gram‐negative bacteria. These genes form a secretion and delivery mechanism for several different virulence factors, including hemophores, proteases, and lipases to target cells (Masi & Wandersman, 2010). The *rtxA1* toxin is known to be associated with Type 1 secretion models (Lee et al., 2008). Within all three genomes examined, multiple genes related to the T1SS secreted agglutinin RTX; however, WR2‐BW and ATCC 27562^T^ only contained two copies of the three genes that made up the system and did not contain *rtxA1*. One hypothesis for reduced virulence may be a reduction in the delivery of the RTX toxin into the host organism by strains lacking this gene compared to JY1701.

The wzxE protein is another interesting candidate for virulence. The less virulent *V. vulnificus* Aug WR2‐BW contains a similar *wzx* gene within a PAI (Klein et al., 2018); however, it is a variant of the *wzxE* gene encoding the O‐antigen flippase, rather than *wzxE*. This family of genes is considered to be a virulence‐*associated* factor, as host cell damage is not directly caused, but it does contribute to pathogenesis and aids in infection. A related gene, belonging to the oligosaccharide flippase family based on the NCBI Protein Blast score (99%), was found in JY1701. The most prominent component of the outer membrane of Gram‐negative bacteria, lipopolysaccharide (LPS), is known to induce fevers (Jones & Oliver, 2009; McPherson et al., 1991). Genes that fall under this category of translocation of lipid‐linked oligosaccharides are used in activities, such as cell wall construction, polysaccharide synthesis, and protein glycosylation; however, the wzx/wzy pathway remains partially undefined and research is still needed to fully understand this pathway and all its functions (Hong et al., 2017).

The virulence‐associated E proteins belong to the family of proteins that contain a p‐loop motif, or phosphate‐binding loops. Virulence‐associated proteins have been identified in other microorganisms, including *Streptococcus* and *Rhodococcus* species (Ji et al., 2016; Okoko et al., 2015). Mice that were exposed to a strain of *Streptococcus suis* serotype 2 that had a functional copy of the virulence‐associated E protein (vapE) exhibited more severe symptoms, which included behavioral symptoms of apathy and depression, along with anorexia, fever, emaciation, and neural disorders. These mice succumbed to infection within 2 days. Mice exposed to a *vapE* mutant exhibited less severe clinical symptoms and all recovered within a week (Ji et al., 2016). The role that *vapE* plays in pathogenicity is poorly understood, but these trials indicate a role for *vapE* in the virulence of this species of *Streptococcus*. Because of *vapE*'s undefined overall function and role in pathogenicity mechanisms, it is unclear how its role in *V. vulnificus* virulence is involved, but strong evidence from here and others suggests it may play a role.

The putative integrase may be involved in virulence differently, as a site of recombination and integration of foreign DNA (Hacker & Carniel, 2001; Hacker & Kaper, 2000). *Vibrio cholerae*, a close relative of *V. vulnificus*, also has putative integrase genes, the accessory colonization factor (ACF), and the toxin‐coregulated pilus (TCP) (Kovach et al., 1996). ACF and TCP are involved in the colonization of host cells and greatly aid in the ability to successfully colonize the host intestinal epithelial tissue (Kovach et al., 1996). These genes may have been obtained by *Vibrio cholerae* via horizontal gene transfer or through other mobile genetic elements, thus it is important to note that this putative integrase gene is present in strain JY1701 and not in the other two strains, suggesting that *V. vulnificus* JY1701 may be able to obtain other PAIs, virulence factors, and other foreign DNA from other organisms at a much faster rate.

Finally, we have observed that strains with high virulence in the fish also frequently deplete the fish of blood, indicating that it may serve as a nutritional source of iron. *V. vulnificus* strains are known to possess siderophores, or iron acquisition molecules, which are low‐molecular‐weight chelators that bind iron and are then returned and brought back into the cells (León‐Sicairos et al., 2015, Simpson & Oliver, 1983). In many cases, blood serum iron is unavailable to microorganisms due to inhibitory effects, which creates an iron deprivation for microorganisms while in the bloodstream (Weinberg et al., 1978). When iron‐containing compounds that are more biologically available have been injected directly into the blood during animal infection models, there has been an increase in microbial numbers (Holbein et al., 1981; Kochan et al., 1977). Thus, the ability of *V. vulnificus* to produce siderophores may provide an important but under‐recognized virulence mechanism to provide the high levels of iron required for sustained survival. Additionally, *V. vulnificus* can use exogenous siderophores produced by other bacterial species (Alice et al., 2008; Aso et al., 2002; Barnes et al., 2020). Due to the nature of the IP injection we used in this study, there is a possibility that some natural skin microbiota of the zebrafish were introduced along with the *Vibrio* bacteria during injection. These include *Enterobacteriaceae* sp, *Flavobacterium* sp, and *Sphingobacteriales sp*, among others, which are also known to produce siderophores (Nakatani & Hori, 2021). Although the skin bioburden would be significantly less than the introduced *V. vulnificus* dose, these strains may also play a role in iron acquisition by *V. vulnificus*. Consequently, iron acquisition in *V. vulnificus* may play an important role in sustaining an infection. Continued examination into the siderophore usage between the virulent strains versus less virulent strains may provide further evidence for the importance of this mechanism.

Here, we have identified and characterized several environmental isolates of *V. vulnificus* with a range of virulence in vivo*.* Our work demonstrates the importance of understanding that virulence between different environmental strains varies greatly and cannot accurately be predicted based on genotype alone. The pathogenicity mechanism for this organism remains elusive and hidden in the genome, requiring full genome sequencing of more environmental strains to aptly determine which genes may be involved in virulence that remain to be identified.

## AUTHOR CONTRIBUTIONS

**Shannon Pipes**: Conceptualization; methodology; investigation; formal analysis; validation; writing—original draft; writing—review and editing; funding acquisition. **Charles R. Lovell**: Funding acquisition; supervision. **Katie L. Kathrein**: Conceptualization; methodology; investigation; formal analysis; supervision; writing—review and editing; methodology; funding acquisition.

## CONFLICT OF INTEREST STATEMENT

None declared.

## ETHICS STATEMENT

The Institutional Animal Care and Use Committee (IACUC) at the University of South Carolina approved protocols used for zebrafish husbandry.

## Data Availability

Genomes analyzed are available in NCBI GenBank at https://www.ncbi.nlm.nih.gov/genbank/ using accession numbers: AFSY00000000 (*V. vulnificus* JY1701), NZ_AMQV00000000 (*V. vulnificus* ATCC 27562^T^), and GCA_003798485.1 (*V. vulnificus* WR2‐BW).
